# Taking patient and public involvement online: qualitative evaluation of an online forum for palliative care and rehabilitation research

**DOI:** 10.1186/s40900-018-0097-z

**Published:** 2018-05-01

**Authors:** Lisa Jane Brighton, Sophie Pask, Hamid Benalia, Sylvia Bailey, Marion Sumerfield, Jana Witt, Susanne de Wolf-Linder, Simon Noah Etkind, Fliss E. M. Murtagh, Jonathan Koffman, Catherine J. Evans

**Affiliations:** 1 Cicely Saunders Institute of Palliative Care, Policy and Rehabilitation, King’s College London, Bessemer Road, London, SE5 9PJ UK; 2 Patient/Carer Representative, Cicely Saunders Institute of Palliative Care, Policy and Rehabilitation, King’s College London, London, UK; 3 Cancer Research UK, Angel Building, 407 St John Street, London, UK; 4 Zurich University of Applied Sciences, School of Health Professions, Institute of Nursing, Winterthur, Switzerland; 5 Wolfson Palliative Care Research Centre, Hull York Medical School, University of Hull, Hull, UK; 6 Sussex Community NHS Foundation Trust, Trust HQ Brighton General Hospital, Brighton, Elm Gove UK

**Keywords:** Patient participation, Patient engagement, Online systems, Palliative care, Rehabilitation, Patient and public involvement, Service user involvement, Online forum

## Abstract

**Plain English summary:**

Patient and public involvement (PPI) is increasingly recognised as important in research. Most PPI takes place face-to-face, but this can be difficult for people who are unwell or have caring responsibilities. As these challenges are particularly common in palliative care and rehabilitation research, we developed an online forum for PPI: www.csipublicinvolvement.co.uk. In this study, we explored how well the online forum worked, if it is a suitable method for PPI, and how PPI members and researchers reacted to using it. We used an existing theory about online interventions to help choose the ‘right’ questions to ask participants. We invited PPI members and researchers who had used the online forum to participate in focus groups, and identified the most important themes discussed. Within this study, PPI members have helped with the interview questions, analysis, and write up. Overall, four PPI members and five researchers participated in the focus groups. Participants felt the online forum worked well and had multiple benefits. From the discussions, we identified four key questions to consider when developing online methods for PPI: how does the forum work, how does it engage people, how does it empower people, and what is the impact? Participants suggested the forum could be improved by being more PPI and less researcher focused. We conclude that when developing online methods of PPI, a functioning forum is not enough: it also needs to be engaging and empowering to have an impact. Future work can use these four domains when developing their own online PPI methods.

**Abstract:**

**Background:**

Patient and public involvement (PPI) in research is increasingly recognised as important. Most PPI activities take place face-to-face, yet this can be difficult for people with ill health or caring responsibilities, and may exclude people from hard-to-reach populations (e.g. living in vulnerable social circumstances and/or remote geographical locations). These challenges are particularly pertinent in palliative care and rehabilitation research where people often live with, or care for someone with, advanced illness. In response to this, we aimed to test the functionality, feasibility, and acceptability of an online forum for PPI for palliative care and rehabilitation research (www.csipublicinvolvement.co.uk).

**Methods:**

We conducted separate focus groups with PPI members and researchers who had used the online forum. Data collection was underpinned by DeLone and Mclean’s model of information systems success. Focus groups were recorded, transcribed, and analysed using inductive thematic analysis. Dual coding by two authors ensured rigour, and attention was paid to divergent cases.

**Results:**

Four PPI members and five researchers participated in the focus groups (two PPI focus groups, one researcher focus group). The online forum was perceived as functional, feasible, and acceptable. Our analysis identified four key questions to consider when developing online methods for PPI: (1) how does the forum work, (2) how does it engage people, (3) how does it empower people, and (4) what is the impact? PPI members felt that the online forum was too researcher led, and needed to be more PPI focussed.

**Conclusions:**

When developing online methods of PPI, a functioning forum is not enough: it also needs to be engaging and empowering to have an impact. To optimise online involvement, future work should refer to these four domains and balance the needs of researchers and PPI members.

**Electronic supplementary material:**

The online version of this article (10.1186/s40900-018-0097-z) contains supplementary material, which is available to authorized users.

## Background

The importance of Patient and Public Involvement (PPI) throughout the research process is increasingly recognised [1, 2]. In addition to meeting ethical principles of not conducting research about people without their input [3], it informs research priority setting [4], provides fresh insight regarding study design and outcomes [5], has increasingly become a requirement when applying for research grants [6], and gives opportunities for PPI members to gain confidence and knowledge in research [7]. PPI is particularly pertinent to conducting research that is mindful of the goals and philosophies of palliative care and rehabilitation: to improve quality of life for the patient and the family [8], optimise functioning, and reduce disability [9]. This also requires research designs to be acceptable for people living with advanced disease, and their loved ones [10]. Large organisations within palliative care now promote PPI in research and practice [11, 12].

There is uncertainty regarding how best to do PPI [13], and how to support and involve a diversity of patients and public in palliative care and rehabilitation research. Most PPI activities take place face-to-face, yet this presents several challenges [14]. Firstly, minority and hard-to-reach populations (e.g. those living in vulnerable social circumstances and/or in remote geographical locations) are often not adequately represented [15, 16]. With evidence of inequities in access to palliative care [17], research to address this must be accompanied by PPI that represent the diversity in our communities. Secondly, many people living with advanced conditions, or caring for a person, find it difficult to attend face-to-face PPI. For patients, fatigue and mobility restrictions present challenges [18], and the importance of time when living with a life limiting condition. For carers (family or friends), work and/or family or caregiving commitments, or ill health, limit time available to support research. Their priorities are often to care for their loved one and being “on call” for them, limiting time to attend meetings in person. Finally, a limited budget for PPI puts constraints on the frequency of meetings, and the number of people able to attend. This makes it difficult to maintain collaborative relationships and an ongoing dialogue throughout the duration of projects.

**Fig. 1:**
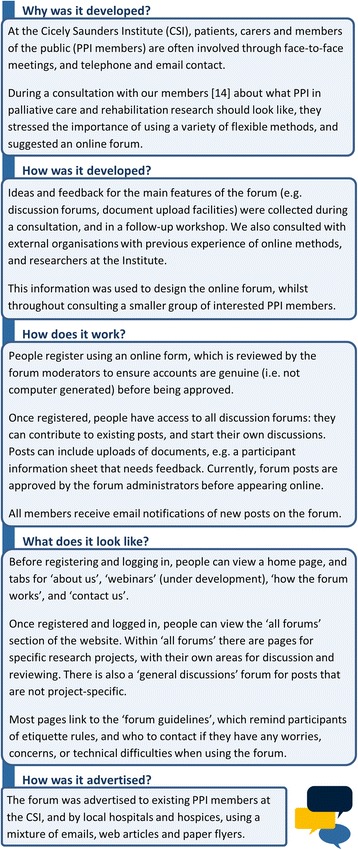
Details of the CSI Public Involvement Online Forum

In response to such challenges, virtual methods have been suggested by PPI members and researchers as a supplement to face-to-face PPI [14, 19]. We therefore developed and launched an online forum to improve engagement and involvement in palliative care and rehabilitation research: the first of its kind in these fields. The online forum, www.csipublicinvolvement.co.uk, is hosted on a dedicated ‘Moodle’ platform: a platform used by many UK universities to create personalised learning environments. Details on how it was developed and how it works are shown in Fig. 1.

Evaluation of new approaches is essential to understand impact on research work, and inform further development and refinement of this novel PPI method, in line with the needs and preferences of PPI members and researchers. Furthermore, evaluation of the online forum and dissemination will inform the wider field of PPI in health research. This study aimed to assess the functionality, feasibility and acceptability of an online forum for PPI in palliative care and rehabilitation research, from the perspectives of PPI members and researchers.

## Methods

### Design

Qualitative focus group study, reported in line with COREQ [20] (Additional file 1: Table S1).

### Setting

A palliative care and rehabilitation research institute in South London, UK.

### Sampling and recruitment

All registered online forum users were invited to participate in the focus groups. This includes researchers from the Cicely Saunders Institute, and PPI members with a range of experience with palliative care and/or rehabilitation clinical practice and research. The focus groups were advertised by email, and reminders were displayed on the front page of the online forum.

### Data collection

To ensure comprehensiveness, our evaluation was underpinned by the updated DeLone and McLean Model of Information Systems Success; a well-established and highly-used model in the information systems literature [21]. This model suggests the success of any information system is dependent on five inter-related system factors: information quality, system quality, and service quality (all elements of functionality), intention to use and actual use (feasibility), and user satisfaction (acceptability). We structured our focus group topic guides to ensure we captured each of these components. In addition, prior to the focus groups, we administered a brief online survey to all online forum participants. The topic guides were then revised to address issues raised in the online survey (online Additional file 1: S2 and S3).

Semi-structured face-to-face focus groups were conducted separately with PPI members and researchers, led by researchers experienced in qualitative research, palliative care, and PPI [LB, SP, SE]. These researchers were actively involved in supporting PPI activity at their research institute and had contributed to developing the online forum. Researcher participants and some of the PPI participants were previously known to the researchers. Field notes were taken by one of the researchers, and each focus group was recorded digitally and transcribed verbatim. All focus groups were held at the research institute. Travel expenses were reimbursed and refreshments provided. In this preliminary research, the number of focus groups was limited by available funding. It was therefore not possible to collect data up until thematic saturation was reached. Demographic data were obtained from participants’ online forum registration details.

### Analysis

Focus group data were analysed inductively and thematically [22] through five stages: (1) familiarisation with the data, (2) preliminary coding frame generated, (3) coding frame revised with input of project advisory group, (4) coding frame applied to all data, (5) dual coding to check reliability of application [LB, SP] [23]. Attention was paid to divergent cases throughout analysis [24]. Initially, deductive coding using the Model of Information Systems Success had been planned (i.e. to create a coding frame based on their categories of ‘information quality’, ‘system quality’ etc., and apply this to our data). However, as we became familiar with the data, we realised these categories did not fully describe what was said in the focus groups. We therefore decided to use an inductive approach instead (i.e. using the focus group data itself as a basis for our coding frame). Qualitative analysis was managed in QSR NVivo 10 [25].

### Ethical approval

Ethical approval was received from King’s College London’s Research Ethics Committee (Reference: LRS15/162623). Focus group participants gave informed written consent.

### Patient and public involvement in this study

PPI members were involved in developing the interview topic guide, feeding back on the analysis and interpretation, revising the final paper, and presenting preliminary findings at local and national meetings. The GRIPP-SF [26] checklist has been used to summarise PPI in the current study in more detail, including reflections for future work (Additional file 1: Table S4).

## Results

Of 63 online forum users invited, 13 registered for focus groups, and 9 took part. Four PPI participants cancelled on the day of the focus groups due to unforeseen circumstances. Three focus groups were held: one with researchers, and two with PPI members. Focus groups lasted for median 77 min (range 60 to 78 min). PPI member and researcher participant characteristics are shown in Table 1.

**Table 1:** PPI member and researcher participant characteristics

	PPI (*n* = 4)	Researcher (*n* = 5)
PPI type		
Patient	0	–
Patient & relative/carer	1	–
Relative/Carer	2	–
Interested public	1	–
Gender		
Female	3	4
Male	1	1
Ethnicity		
Black African	0	1
Black Caribbean	0	0
White British	2	4
White Other	2	0
Age		
Mean (SD)	63.3 (3.2)	36.0 (7.1)
Previous PPI experience		
None	0	1
6 months to 2 years	0	3
Over 2 years	4	1
Has a disability		
Yes	3	0
No	1	5
Location		
London	0	–
Outside London	4	–

**Fig. 2:**
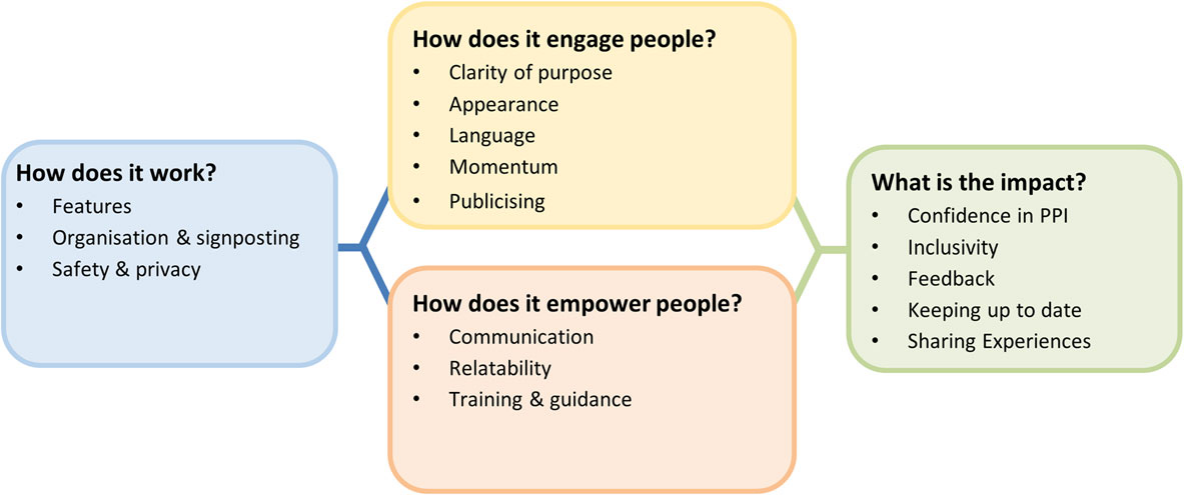
Model of successful online patient and public involvement

Four core themes were identified, and displayed in our theoretical model (Fig. 2): How does the forum work, how does it engage people, how does it empower people, and what is the impact? A full coding tree can be found in Additional file 1: Table S5.

## How does the forum work?

Participants identified three key aspects they felt contributed towards making the forum functional.

### Features

An important feature for all groups were the email notifications that update users on forum activity. Some PPI members found it difficult to determine the context in which the comments were made, but appreciated that they could see the whole thread of conversation on the forum. Both groups agreed that a weekly update would be useful.

*“I was expecting, I suppose, an email or a generic email saying, ‘We're looking at x, have a look for us.’ That hasn't come through. What I seem to get is, I don't get the question or the proposal or the project, I just get other people's comments. So you're getting one side of it”.* PPI Member 3, Focus Group 2.

PPI members were very positive about the idea of hosting webinars, as this may generate interest in the online forum. A few participants suggested that webinar themes could be led by PPIs to reflect their training needs and interests.

*“That would be incredibly useful because I know that you've held some training sessions here, I seem to think, which, if you live a distance away, that's not easy to come all this way for an hour's tutorial or L&D session or whatever. So I think the idea of the webinars is increasing in popularity. As for topics, I think that's your first post on the online forum.”* PPI Member 4, Focus Group 2.

Researchers and PPI members felt that the forum was a useful place to facilitate networking between PPI members and obtain peer support in between face-to-face meetings.

*“I know it's another bonus from it isn't it - when our group meet as a group they clearly really enjoy all coming together … and you want that sort of - as the forum - to try and facilitate that further.”* Researcher 5.

Researchers and PPI members highlighted a number of additional features that would be useful to include on the online forum, including: a search function, a scrap book to compile posts of interest, integration of the Institutes Twitter feed, space for PPI members to blog, and a video on PPI members’ experiences of using the forum. Some participants felt that it would be useful to link to other sites from the forum, including resources about PPI and e-learning about research.

### Organisation and signposting

All groups expressed that the online forum was easy to use. Researchers felt it was easier to navigate for researchers than PPI members, for example due to use of project names:

*“I think it was easier for us as researchers, maybe because some of it is structured by project title. So it's like a project acronym, this, that and the other. And I just wondered if I was somebody who was a representative coming to it cold, whether I would actually know what those things were.”* Researcher 3.

In support of this, PPI members described how they felt unsure of how to interact with the project-specific pages. They suggested that themed topics would be more useful for navigation. This could include key topics in palliative care and rehabilitation (e.g. older people, advance care planning).

*“If you wanted to put up the tabs that said ‘Feeling safe’, I would go to that, as a member of the public, or something else, like- not ‘Advance care planning’, but ‘Are you ready? Be ready for it’*”. PPI Member 1, Focus Group 1.

However, some PPI members suggested that ability to navigate the online forum would be improved through familiarity. Overall, PPI members felt that any future changes should be guided by patient, carers and members of the public.

### Safety and privacy

Safety was a big priority for PPI members. They felt that having to log into the forum demonstrated the site was safe:

*“The only thing I would say about logging in is, because you are empowering people and saying, ‘This is a safe site to be on.”* PPI Member 2, Focus Group 1.

However, as a new website, they felt it was important to emphasise safety in text on the forum also. Members of both the PPI and research focus groups suggested the ability to login via Facebook or Twitter would make remembering passwords easier:

*“Yes. It’s a bit of a nuisance, though. Because I’ve got the ease of clocking into Facebook and Twitter, which is always on. I’ve got to think, ‘What’s my password?’ And I’ve forgotten it again”.* PPI Member 1, Focus Group 1.

They also felt strongly that email addresses should not be accessible to other members on the forum.

## How does it engage people?

Feasibility of the online forum as a method of PPI depended heavily on its ability to engage researchers and PPI members, and this depended on multiple factors:

### Clarity of purpose

PPI members highlighted that the purpose was not clear from the home page, making it less engaging for new users. They were also unsure if discussions could be led by PPI members, as well as researchers:

*“I think, for yourselves, it's whether, is this going to be just a forum where researchers post and ask questions and we give feedback? Or, is it where we can post things to prompt?”* PPI Member 4, Focus Group 2.

Some researchers highlighted that they weren’t sure how else they could use the forum other than for document feedback.

*“I think for me it's knowing what - apart from the obvious check out this document, what do you think? I think there are other questions that I might want to ask a person and I think it's knowing how free range we can be with the types of questions.”* Researcher 4.

### Appearance

Participants felt the appearance could be more engaging. The design had a very academic feel, and would benefit from the use of brighter colours:

PPI Member 4: *“Little bit more colourful because it is very researchery, very…”.*

PPI Member 3: *“It's very institutional.”* Focus group 2.

PPI members felt that there was nothing in the design to say “this is for you”, and all felt that the appearance was researcher-led rather than PPI-led:

*“It kind of seemed fairly, you know, institutionally led rather than patient-friendly led, the interface, the way it looks.”* Researcher 4.

### Language

All felt that the language on the forum should be clear and personal. PPI members emphasised that acronyms and unfamiliar project names should be avoided, as they are not as engaging:

*“We want your involvement in, dash, Feeling Safe, C-Change, dash, IARE-II Study.’ It needs to be more accessible, doesn’t it?”* PPI Member 1, Focus Group 1.

Researchers agreed and felt that they often get immersed in research terminology and forget this is not always accessible.

*“I think you sort of get immersed into the terminologies and forget and everything seems straight forward.”* Researcher 2.

Language was also described as important in illustrating that the online forum exists, so that they can share their views and shape research.

PPI member 2: “*Yes. So, somehow, we’ve got to get this so that people will meet this, find it through a search engine, which is where most people will get it from, won’t they?”*

Interviewer 1: “*Yes.”*

PPI member 2: “*And look at it and say, ‘Hey, this looks good. I want to be in that.’ And I think we need to re-look at the language, because initially, ‘We really need you. We need your opinions. Can you help us with this?’”* Focus Group 1.

### Momentum

Everyone felt that the forum would become more engaging as it gathered more users and momentum. PPI members emphasised it needs to be engaging enough to compete with other commitments:

*“I’m involved in three or four research projects around [city], which is where I live … So, I tend to get hooked on the latest thing. I keep saying, ‘When I’ve got a spare half-hour, I’ll get back on the site and see what’s going on.’ And I haven’t had the spare half-hour…”.* PPI Member 2, Focus Group 1.

PPI members felt that they were primarily responding to prompts from researchers, and there were often peaks and troughs in activity. However, all groups recognised that the online forum is still in the early stages of development.

*“You have a flurry of emails, it can be two or three, every two or three hours and then, suddenly, nothing for two weeks and the same again. You're losing the momentum and you don't know what these emails are talking about.”* PPI Member 3, Focus Group 2.

One PPI member discussed structures used by other social media sites and how having a more conversational tone encouraged activity.

*“I think the reading of it does encourage you, because that’s the problem, and the beauty, and the difficulty with Twitter. The beauty is that you can read, and can react very quickly and effectively”.* PPI Member 1, Focus Group 1.

### Publicising

PPI members felt publicising to encourage membership would help give the forum more momentum. They recommended the forum should be listed on search engines and advertised on social media, and through communities, PPI groups and charities:

*“I think once you’ve got it up and running, the next question is, how do you publicise it, and where do you publicise it? I think it needs a much bigger coverage. You can’t just leave it sitting on the Internet and say, ‘People will find it’, because they won’t, necessarily.”* PPI Member 2, Focus Group 1.

*“I mean, I think the ‘why we do research’ hashtag would be really good, because that has had millions of clicks in less than two years.”* PPI Member 1, Focus Group 1.

Participants queried how we would include minority and hard-to-reach groups, specifically people with limited experience or interest in PPI in palliative care and rehabilitation research, and/or those who may struggle with computer literacy. Some suggested that a promotional video with current members would help to promote interest in the forum:

*“Whether you could have just a small video piece from your PPI reps, just talking to say, ‘This is how we find using the forum. Don't be put off by it.’ Almost like a bit of a sales pitch so ordinary folk that aren't academics, you know, just saying, ‘We found this useful because if you're passionate about end-of-life care and just…’”.* PPI Member 1, Focus Group 1.

One researcher explained that they wanted to see the forum before they publicised it to their contacts, to ensure that it was suitable to use:

*“I wanted to have a look for myself before I recommended it to other people to use.” Researcher* 5.

## How does it empower people?

Feasibility of the online forum was dependent on PPI members and researchers feeling empowered to participate. Three key aspects contributed to this:

### Communication

Communication between PPI members and researchers on the forum was critical to empowerment, and an area in need of improvement:

*“I think we’ve got a communication problem here, for a start, and I think it’s partially about empowerment.”* PPI Member 2, Focus Group 1.

Although responses to posts were prompt and polite, PPI members felt initial posts need to make participants to feel at ease. Part of this was about clarity and use of plain English:

*“It's definitely got to be about the Plain English, most definitely, keeping it - especially if we're wanting to extend out, keeping it simple. I torment [researcher] a lot when I look at their summary and there's paragraphs and paragraphs and I think, ‘Oh that could be said in, certainly in sentences instead.”* PPI Member 4, Focus Group 2.

The researchers also acknowledged that they could improve how they had written their posts. However, they were not sure how best to communicate in ways that generate and maintain discussions:

*“I think one of the things that I found hard was sort of generating discussion. And partly it might be the way that I'm writing things”.* Researcher 3.

As there is something exposing about posting on the forum, PPI members suggested posts should be phrased in an encouraging way, making the value of PPI member input clear. For example, one PPI member suggested it should read in a way that says, *“We need your help, and we can’t do this without you.”* (PPI member 1, Focus Group 1).

All felt PPI forum members need to be more empowered to start conversations on the forum.

### Relatability

Relatability refers to how people feel they connect with the forum, and other forum participants. This overlapped with communication on the forum, and how when done badly this can make people feel they’re not the right person to contribute. Instead, forum members need to feel at home:

*“It’s like sitting in a comfy armchair. ‘We’re okay. We’re with somebody who talks our language, understands the way we feel.’ And you’ve got to create that sort of ambience to make it work, I think.”* PPI Member 2, Focus Group 1.

PPI members explained that rather than more intimidating pictures of academics, it would be useful to use natural pictures of day-to-day activities that people could relate to:

*“Maybe just doing ordinary everyday things, if you have someone pushing a shopping trolley or someone on the bus. You know what I mean, rather than… They do look as if you would have the hospital trust's - this is our board.”* PPI Member 3, Focus Group 2.

They suggested sharing experiences of PPI members when they first joined the forum. This could empower new members by acknowledging potential anxieties and how others have overcome these.

Due to the difficulties of building relationships online, the researchers also suggested knowing more about each other may be helpful. One suggested having short personal profiles with pictures, particularly to make the researchers seem less like *“faceless researchers who use research language”* (Researcher 4).

### Training and guidance

Training and guidance also contributed to empowering PPI members and researchers to use the online forum. Although most participants did not refer to the guidance and learnt as they used it, they noted the importance of having this support available for people who are less confident with technology. This could include videos, as they may be more user-friendly than a written document.

Researchers felt more guidance was needed to clarify the different things that the online forum could be used for, and on how to write and respond to posts:

*“Yes, I think I had a bit of anxiety and I came to speak to [the forum administrator] about like, ‘Is this response okay?".* Researcher 1.

Over time, being able to see how the forum has been used and how posts have been written previously will also contribute to this.

PPI members felt that integrating training in research methods into the online forum would be valuable. This would help empower PPI members to post their own ideas and discussion points:

*“That's perhaps something you could work on because I don't have an academic background at all, I come in and I work on a specific thing that I think, ‘Oh I've got some experience or knowledge of that.’ That is maybe a learning and development side that you could give us some help with because it's quite a big confidence thing to suddenly expose yourself on the net there by saying, ‘I’ve had this idea for a research proposal.’ Then everybody's going, ‘Ha, ha, ha. That's just ludicrous’. So maybe just a little bit of support”.* PPI Member 4, Focus Group 2.

Webinars would be potentially helpful here, as long as there is clear guidance on how to use this function.

## What is the impact?

Participants described the overall acceptability of the online forum in terms of both benefits and challenges:

### Confidence in PPI

Researchers described the online forum as an acceptable method of PPI, and how they intended to use it again in the future:

*“I'm thinking - because I've got ethics next week, I'm thinking if ethics come up with any issues that I would - I'm looking forward to having the forum to go to”* Researcher 4.

The ease of using the online forum raised concerns around potential tokenistic use by researchers:

*“I think it's important that people realise that it's not just you put a post in the forum and somebody replies and then you put in your grant that you've done some PPI, because you have to do more than that for it to be meaningful, but I think it's a great place to start.”* Researcher 3.

However, it was also acknowledged that using the forum can increase researchers’ confidence in their ability to involve PPI members:

*“I felt a bit more confident that I'd made further attempt to engage members of the public, because it's quite important for my research that I do that”.* Researcher 2.

### Inclusivity

PPI members and researchers emphasised the benefit of an online forum being more inclusive. This includes people who have caring responsibilities or are unwell, and those who live further away:

*“There comes a point where you are losing your ability to influence everything else, where you can still take control over some things by having an input into that. And when you can’t move, and you can’t decide what you’re going to eat, or what you’re going to wear, that gives you a bit more control”.* PPI Member 2, Focus Group 1.

*“So, maybe that’s another hook there. You can be anywhere. This is across the world, in a way”… “This is not just for people who live in London.”* PPI Member 1, Focus Group 1.

Researchers had concerns about levels of IT ability and how that would impact on inclusivity:

*“I help my aunt a lot at the moment, she's 87, using the internet, because so many things are online now and I think we take for granted that you know how to read a screen. And you know what different buttons mean and you know what tabs are and things like that. And she looks at it and she's totally lost.” ... “we potentially exclude those people”*. Researcher 4.

However, PPI members felt that as long as help is available, this should not be a main concern:

*“My mum's 83 and very competent, probably more so, in some respects, than I am on certain things. I don't think you should worry about being too simplistic and then people can opt into whether they need like a help section”.* PPI Member 4, Focus Group 2.

It was noted that some would still prefer face-to-face methods, and for some aspects of research (e.g. competitive grant applications) the inclusive nature of the forum makes it less suitable. Participants acknowledged it was important to be flexible to the needs of the PPI member, the researcher, and/or the project, and using the online forum accordingly alongside other PPI methods.

*“And I suppose you just have to be flexible don't you? I just think with some people we always have to print documents out for and post them isn't it, because they're not confident about using an email. So in the same way that you're working away to enable somebody to take part”.* Researcher 5.

### Feedback

All emphasised that the online forum provided a space to ask for, and give feedback on, elements of research. Researchers talked about using the forum to discuss topics relevant to their study, and specific study documents (e.g. participant information sheets).

*“[I was] genuinely interested to hear whether they got the content of my study or not. And also whether the patient facing [materials], like with [Researcher 2], whether they were comprehensible and meaningful.”* Researcher 5.

Researchers also used the forum to offer longer-term opportunities for feedback by advertising project advisory roles. They felt that the level of feedback would improve as the forum grew. PPI members noted that there weren’t many posts to provide feedback on so far.

One researcher raised their concerns about empowering participants to give critical feedback, asking *“how easy would it be for a lay member to be critical on the forum?”* (Researcher 4). Another researcher noted however that this may be a particular benefit of feeding back online:

*“That's probably one thing that you get more from out of the online form than in face-to-face because I think it probably is a bit harder to sit there in front of someone and say, ‘Your information sheet is really rubbish’".* Researcher 5.

### Keeping up-to-date

The online forum created a space where people can stay up-to-date with research at the Institute. One PPI member noted how they look at it *“occasionally, just to see what’s going on”* (Patient Representative 1), whilst another liked the opportunity to learn about and contribute to a broader range of projects:

*“For me, joining the forum was an opportunity to have a look at the broad spectrum of what was happening and the opportunity to be able to contribute to other things rather than just the set projects I was involved in”.* PPI Member 4, Focus Group 1.

Researchers also appreciated the ease with which they could use the forum to keep people up-to-date with their research:

*“I think if we found that the group was really big and people outside had an interest in our study and we could communicate with a group of interested public members, that way, we could post things about our study to sort of keep them updated”.* Researcher 1.

### Sharing experiences

The forum was also seen as providing a space for people to share their personal experiences, particularly by PPI members:

*“I think, increasingly, as you’re becoming more and more unwell, and you’re involved with caring for someone, or you yourself are dying, you’ve got to have access to something where you can share things.”* PPI Member 1, Focus Group 1.

One participant noted how this needs clear guidance in order to maintain anonymity of specific people or services that are referred to within posts:

*“As PPI members we could do with a little bit of understanding the politics, sometimes, of what we should and shouldn't say. Something as simple as a ‘do and don'ts’, kind of thing.”* PPI Member 4, Focus Group 2.

## Discussion

Overall the online forum was functional, feasible, and acceptable to PPI members and researchers, but was currently biased towards a researcher-focused design. We identified four key questions to consider when developing and using online methods for PPI: (1) how does it work, (2) how does it engage people, (3) how does it empower people, and (4) what is the impact? The forum needs to be safe for the PPI members to share experiences and thoughts, with website features to support the intended functions (e.g. sharing documents) and easy-to-follow organisation and signposting. However, this alone is not enough. An engaging forum must be aesthetically pleasing and easy to understand, with a clear purpose for joining. It also requires a critical mass of users to help build and maintain momentum, which can be achieved by appealing to users via multiple avenues of publicity. In addition, participants must be empowered to contribute to discussions. This can be supported through training and guidance, but is particularly influenced by how people communicate within, and relate to, the forum. The perceived impact of the forum on research is increasing researchers’ confidence in engaging with PPI, and enabling and widening access to involvement in research beyond people attending face-to-face meetings. However, participants noted that this would not benefit all hard-to-reach groups (e.g. people who are not computer literature), and future work is needed to address this, and the extent to which this method engages people from minority groups. The online forum also provides a dedicated space for sharing experiences, and providing feedback on, and keep up-to-date with, various elements of research. However, future improvements need to ensure the forum becomes less researcher-focused, and more PPI focused, across these domains.

Whilst others have developed online spaces for information provision [27] and sharing experiences [28, 29], this is the first dedicated online forum with two-way dialogue between PPI members and researchers in palliative care and rehabilitation. Our evaluation builds on previous piloting of online methods to engage with people with motor neurone disease at our Institute [18], where despite concerns of the effects of this condition on feasibility, many were able to actively engage. It is known that PPI members favour multiple and flexible methods of PPI [13, 14], particularly in palliative care in the context of fluctuating disease burden and caregiving responsibilities [14]. We found that both PPI members and researchers appreciated this additional option for PPI involvement, and saw it as a tool that would work well alongside other PPI methods, such as face-to-face meetings.

Several themes across existing PPI research resonated with our findings. For example, the importance of clarity of roles and contributions [30]: without this the participants felt it would be difficult to engage new members in the online forum. In addition, the importance of levelling the playing field and reducing the perceived gap between PPI members and researchers, particularly through empowerment. This included increasing relatability, and communicating in an encouraging way [13] without unnecessary jargon [30, 31], as suggested in previous work about face-to-face involvement. Collaboration between PPI members and researchers as early as writing forum posts may be helpful here. The strong empowerment component also relates to previous evidence on the challenges of power differentials in PPI work [13, 32]. However, this is the first time these concepts have been identified together in the context of an online forum for PPI members and researchers. This may suggest that broader evidence around optimising PPI continues to apply in a virtual environment, but also that our findings may have relevance beyond online methods.

### Strengths & Limitations

A strength of this study is that it was underpinnned by the updated DeLone and McLean Model of Information Systems Success. This model is not specific to online forums and patient and public involvement, and therefore did not sufficiently represent experiences in our data. However, it provided a structure that ensured we considered all aspects of what makes an information system like our online forum successful – from the technical aspects of how it works, to peoples’ experiences of using it. Moreover, despite small numbers, we obtained rich data and captured both PPI member and researcher perspectives. The impact of PPI is understood to be challenging to measure [33, 34] and as such is often lacking [35]. Although we provide evidence of the perceived impact of the online forum, future work will be needed to see if, for example, perceptions of increased diversity are reflected in the forum membership and to investigate the impact of the forum on the research conducted.

A limitation of our study is the volunteer sample: views of those who did not respond to the study invitation may differ to those who took part. For example, PPI participants were typically those with some experience in this field; meaning we may understand less about the experience of the forum for those who are new to palliative care and rehabilitation. Furthermore, the face-to-face focus groups may have been challenging for some online forum members to attend. However, focus groups were deemed the best method for exploring participants reactions to the forum in depth, in a way that participants could build on each other’s ideas throughout the discussions [36]. Importantly, this method followed an online survey, to ensure the views of those unable to attend focus groups were still included in the evaluation. As some participants have long-standing relationships with the interviewers, they may have felt hesitant to share negative feedback. However, the interviewers made sure to encourage participants to share their perspectives on both the positive and negative aspects of the online forum, and both types of comments were evident in the data. Additionally, due to the resource restrictions on this preliminary work, and last-minute dropouts from the focus groups, recruitment numbers were low and thematic saturation was not reached. Further evaluation is required to fully understand our emergent theoretical model.

## Conclusion

Our online forum for PPI in palliative care and rehabilitation research was functional, feasible, and acceptable overall for PPI members and for researchers. However, it requires further refinement to better meet the needs of PPI members in addition to researchers. Our theoretical model based on this evaluation demonstrates that it is not enough to have a working online forum; it needs to be engaging and empowering to have impact. As such, those working towards online PPI in health services and research could benefit from considering the four key questions proposed by our model during development and evaluation.

## Additional file

Additional file 1: **S1.** COREQ checklist for qualitative studies, **S2.** Focus group topic guide (PPI members), **S3.** Focus group topic guide (Researchers), **S4.** GRIPP2-SF checklist for PPI in research, **S5.** Coding tree for analysis. (PDF 572 kb)

## Abbreviations

CSI: Cicely Saunders Institute
PPI: Patient and Public Involvement
