# Supplementary material for: Taking patient and public involvement online: qualitative evaluation of an online forum for palliative care and rehabilitation research
Source: Res Involv Engagem. 2018 May 1;4:14. doi: 10.1186/s40900-018-0097-z (PMC5928583; doi:10.1186/s40900-018-0097-z)
Supplement: Supplementary file 1 — S1. COREQ checklist for qualitative studies, S2. Focus group topic guide (PPI members), S3. Focus group topic guide (Researchers), S4. GRIPP2-SF checklist for PPI in research, S5. Coding tree for analysis. (PDF 572 kb) [file 40900_2018_97_MOESM1_ESM.pdf]

Online Supplement:

Taking Patient and Public Involvement Online: Qualitative evaluation of an online forum for palliative care and rehabilitation research

Authors: Brighton, LJ.¶, Pask, S.¶, Benalia, H., Bailey, S., Sumerfield, M., Witt, J., de Wolf-Linder, S., Etkind, S., Murtagh, FEM., Koffman, J., Evans, CJ. (*¶Joint first authors)*

S1: COREQ checklist for qualitative studies

No. Item	Guide questions/description	Reported on Page #
**Domain 1: Research team and reflexivity**		
Personal Characteristics		
1. Inter viewer/facilitator	Which author/s conducted the interview or focus group?	6
2. Credentials	What were the researcher's credentials? E.g. PhD, MD	LB: BSc<br>SP: MSc<br>SE: MD
3. Occupation	What was their occupation at the time of the study?	LB & SP: research assistant<br>SE: PhD fellow
4. Gender	Was the researcher male or female?	LB & SP: Female<br>SE: Male
5. Experience and training	What experience or training did the researcher have?	6
Relationship with participants		
6. Relationship established	Was a relationship established prior to study commencement?	6
7. Participant knowledge of the interviewer	What did the participants know about the researcher? e.g. personal goals, reasons for doing the research	6
8. Interviewer characteristics	What characteristics were reported about the inter viewer/facilitator? e.g. Bias, assumptions, reasons and interests in the research topic	6
**Domain 2: study design**		
Theoretical framework		
9. Methodological orientation and Theory	What methodological orientation was stated to underpin the study? e.g. grounded theory, discourse analysis, ethnography, phenomenology, content analysis	7
Participant selection		
10. Sampling	How were participants selected? e.g. purposive, convenience, consecutive, snowball	6
11. Method of approach	How were participants approached? e.g. face-	6

	to-face, telephone, mail, email	
12. Sample size	How many participants were in the study?	8
13. Non-participation	How many people refused to participate or dropped out? Reasons?	8
Setting		
14. Setting of data collection	Where was the data collected? e.g. home, clinic, workplace	6
15. Presence of non-participants	Was anyone else present besides the participants and researchers?	No
16. Description of sample	What are the important characteristics of the sample? e.g. demographic data, date	8-9
Data collection		
17. Interview guide	Were questions, prompts, guides provided by the authors? Was it pilot tested?	6, Supplements S1 & S2
18. Repeat interviews	Were repeat interviews carried out? If yes, how many?	No
19. Audio/visual recording	Did the research use audio or visual recording to collect the data?	6
20. Field notes	Were field notes made during and/or after the inter view or focus group?	No
21. Duration	What was the duration of the inter views or focus group?	6
22. Data saturation	Was data saturation discussed?	7
23. Transcripts returned	Were transcripts returned to participants for comment and/or correction?	No
**Domain 3: analysis and findings**		
Data analysis		
24. Number of data coders	How many data coders coded the data?	7
25. Description of the coding tree	Did authors provide a description of the coding tree?	10-26, Supplement S5
26. Derivation of themes	Were themes identified in advance or derived from the data?	7
27. Software	What software, if applicable, was used to manage the data?	7
28. Participant checking	Did participants provide feedback on the findings?	No
Reporting		
29. Quotations presented	Were participant quotations presented to illustrate the themes/findings? Was each quotation identified? e.g. participant number	10-26
30. Data and findings consistent	Was there consistency between the data presented and the findings?	10-26
31. Clarity of major themes	Were major themes clearly presented in the findings?	10-26
32. Clarity of minor themes	Is there a description of diverse cases or discussion of minor themes?	10-26

S2: Focus group topic guide (PPI members)

Focus group ground rules & Introduction

- Everything said here is confidential, so please don't repeat to others.
- You are being recorded but this will be transcribed and anonymised.
- If you change your mind about anything you said you can ask us to remove it up to a week after the focus group.
- Try not to talk over each other as it is important to hear everyone's point of view.
- No right or wrong answers - we are keen to know what does and doesn't work.
- This should take about 1 hour, feel free to pop out if needed.
- Fire alarms/toilets

Introductions if needed, and introduce focus group topics

Reasons for joining the forum & Use so far

1. **Why did you join the CSI Public Involvement Forum?** *What was your experience of PPI before this? Did you feel like you had to join?*

Learning to use the forum

2. **What have you used it for so far?** *General discussions? Reviewing study materials? Or observing what's going on? If observing, why do you do this? Has anyone posted a question themselves? If not, why? Would you feel that this is something you could do?*

3. **Overall, how did you find getting started with/learning to use the forum?**
   Did you look at the 'how to' guidance? Did you look at the terms of reference? If so, what did you think, if not, why not? How could this be better?

System quality (show the forum on the screen)

4. **What did you think of the website itself in terms of presentation and layout?** *What do you think of the colours, images, and fonts? Etc. How could this be better?*

5. **What did you think of the website navigation and speed?** *Was it easy or tricky to find your way around? What do you use to navigate (tool bar or buttons)? Was it quick or slow to move between pages? What about posting on discussions? What about viewing or adding documents? How could this be better?(e.g. how should things be organised)*

Information quality

6. **What do you think about the amount and clarity of information on the forum?** *Is it relevant? Is it easy to understand? Is there enough information? Is it clear? How could this be better?*

Service quality

7. **How were the posts from the researchers?** Were they interesting or not so much? What posts did you find more interesting? Were they clear/unclear? Too long or brief? Were there enough or too many? *How did you know when they posted?*

8. **How were the responses from researchers?** Did they respond? Was it a quick response? *Quality of response? How did you know when they responded?*

9. ***How could this be better when interacting with the researchers? (i.e. what should researchers be doing, what could we do the facilitate this on the forum?)***

10. **As the forum administrators, how could we better support you in using the forum?** *Would you like a tutorial video? What else could we do to support you?*

Benefits and challenges of the forum

11. **Overall, what were the challenges of using the forum?** *Did you feel you could respond to posts? Did you remember it was there to use? Did it take a lot of time to use? If so, why? How do these challenges compare to other methods of involvement (e.g. face-to-face)? <u>How could this be better?</u>*

12. **Overall, what were the benefits of using the forum?** *Did you feel that you could contribute/impact the research? Did you learn anything new? Did you connect with new people? How do these benefits compare to other methods of involvement (e.g. face-to-face)? <u>How could this be better?</u>*

Intentions to use in the future

13. **Do you think you will use the forum in the future?** *If so how, if not, why not?*

Final points and close

14. **Is there something else you would like to add before we end the focus group?**

Please remember that if you change your mind about anything you said you can ask us to remove it up to a week after the focus group

S3: Focus group topic guide (Researchers)

Focus group ground rules & Introduction

- Everything said here is confidential, so please don't repeat to others.
- You are being recorded but this will be transcribed and anonymised.
- If you change your mind about anything you said you can ask us to remove it up to a week after the focus group.
- Try not to talk over each other as it is important to hear everyone's point of view.
- No right or wrong answers - we are keen to know what does and doesn't work.
- Should be about 1 hour, feel free to pop out if needed.

Introductions if needed, and introduce focus group topics

Reasons for joining the forum & Use so far

15. **Why did you join the CSI Public Involvement Forum?** *Were abouts in the project were you? What was your experience of PPI before this? Did you feel like you had to join?*

Learning to use the forum

16. **What have you used it for so far?** *General questions? Information sheets? Topic guides?*

17. **Overall, how did you find getting started with/learning to use the forum?** *How could this be better?*

System quality (show the forum on the screen)

18. **What did you think of the website itself in terms of presentation and layout?** *What do you think of the colours, images, and fonts? Etc. How could this be better?*

19. **What did you think of the website navigation and speed?** *Was it easy or tricky to find your way around? Was it quick or slow to move between pages? What about posting on discussions? What about adding documents? How could this be better?*

Information quality

20. **What do you think about the amount and clarity of information on the forum?** *Is it relevant? Is it easy to understand? Is there enough information? Is it clear? How could this be better?*

Service quality

21. **How were the responses from PPI members?** *Was it fast or slow? Too long or too brief? Quality of response? How did you know when they responded? How could this be better?*

22. **As the forum administrators, how could we better support researchers using the forum?** *Did you look at the 'how to' guidance? If so, what did you think, if not, why not? Was it clear what the forum could be used for?*

Benefits and challenges of the forum

23. **Overall, what were the challenges of using the forum?** *Did you struggle to get responses? Did you remember it was there to use? Did it take a lot of time to use? How do these challenges compare to other PPI methods? How could this be better?*

24. **Overall, what were the benefits of using the forum?** *Did it impact on your research? Did you learn anything new? Did you connect with new people? How do these benefits compare to other PPI methods? How could this be better?*

Intentions to use in the future

25. **Do you think you will use the forum in the future?** *If so how, if not, why not?*

Final points and close

26. **Is there something else you would like to add before we end the focus group?**

    Please remember that if you change your mind about anything you said you can ask us to remove it up to a week after the focus group

S4: GRIPP2-SF checklist for PPI in research

**Section and topic**	**Item**
**1: Aim**<br>Report the aim of PPI in the study	To ensure an acceptable and appropriate research process, clear interpretation and appropriate dissemination of the findings.
**2: Methods**<br>Provide a clear description of the methods used for PPI in the study	PPI involvement in this study used a mixture of face-to-face methods (e.g. feedback during Institute PPI strategy meetings) and remote methods (via telephone and email).
**3: Study results**<br>Outcomes—Report the results of PPI in the study, including both positive and negative outcomes	PPI members contributed to the focus group topic guide, revising the manuscript and disseminating preliminary findings. Additionally, feedback was provided on the coding frame but not all members felt able to comment on this.
**4: Discussion and conclusions**<br>Outcomes—Comment on the extent to which PPI influenced the study overall. Describe positive and negative effects	PPI input ensured our topic guides covered all relevant questions, and that our analysis and write-up was not limited to researcher perspectives. This included helpful suggestions for how we can take this work forward in response to our results. Their help with preliminary dissemination has also ensured clarity (particularly in terms of using plain English) and broadened dissemination avenues.
**5: Reflections/critical perspective**<br>Comment critically on the study, reflecting on the things that went well and those that did not, so others can learn from this experience	PPI input on this work was helpful, particularly as this work focuses on creating two-way dialogue between PPI members and researchers. PPI members' comments on the analysis and findings were invaluable. On reflection, we realise that understanding of qualitative methods had been presumed, and that in the future offering additional training in qualitative research methods may be helpful. In addition, it may have also been helpful to train and support our PPI members to co-facilitate the focus groups, and be involved in the earlier stages of coding the data. This may ensure a better balance of researcher and PPI perspectives in future evaluation work.

Table S5: Coding tree for analysis

Themes	Codes	Definitions	Notes
**How does the forum work?**<br><br>The components that contribute to a working forum	**Features**	Thoughts around the current and potential features of the forum (e.g. document sharing, webinars).	"Yes, I think the idea of the webinars, certainly that interests me hugely." (PPI_04)
	**Organisation and signposting**	Finding/getting to where things are on the forum	"I think we take for granted that you know how to read a screen. And you know what different buttons mean and you know what tabs are and things like that. And she looks at it and she's totally lost. She can't read it like we can. So I think that front page and the how people are signposted into the different functions within the forum needs to really - needs to be thought about." (RSR_04)
	**Safety and privacy**	Security of the forum (e.g. safeguarding, bots, trolls, logging in)	"…because Internet security is a very, very big priority at the moment, obviously, if you log in with Facebook or Twitter or those things, you have the password protection actually there. It may not be apparent, but it's just a statement somewhere saying, "This is a safe site." People are very wary of non-safe sites, these days." (PPI_02)
**How does it engage people?**<br><br>Aspects important in engaging users with the forum	**Clarity of purpose**	Clarity of what and who the forum is for	"I don't think there's a clear purpose as to what it's there for." (PPI_04)
	**Appearance**	What the forum looks like	"They're maybe a little bit too old and maybe a little bit too professional. You want brighter colours and some younger people, some mums with kids, you know, sort of… Just to appeal" (PPI_07)
	**Language**	The words used on the forum	"And look at it and say, "Hey, this looks good. I want to be in that." And I think we need to re-look at the language, because initially, "We really need you. We need your opinions. Can you help us with this?"" (PPI_02)
	**Momentum**	The level of activity needed for the forum to be engaging	"I can't say that it's awfully exciting at the moment. I realise that it's a work-in-progress and it's not been going that long" (PPI_04)
	**Publicising**	Comments about raising awareness of the forum	"I think once you've got it up and running, the next question is, how do you publicise it, and where do you publicise it? I think it needs a much bigger coverage. You can't just leave it sitting on the Internet and say, "People will find it," because they won't, necessarily." (PPI_02)

**How does it empower people?**<br><br>Aspects that are important in empowering people to contribute to the forum.	**Communication**	Communication between forum participants	“t’s like sitting in a comfy armchair. “We’re okay. We’re with somebody who talks our language, understands the way we feel.” And you’ve got to create that sort of ambience to make it work, I think.” (PPI_02)
	**Relatability**	Enabling a person to feel that they can connect with the forum and forum participants	“Yes, exactly. So, to me, there’s nothing there that, as an ordinary member of the public- I’m going back seven years, to when I got involved in all of this. There’s nothing there that would say to me, “They really want me.” (PPI_02)
	**Training and guidance**	Training and support in research/PPI, within and beyond the forum	“Do you think people know the research questions? They might understand the issues, but maybe they wouldn’t know what constitutes a research question.” (PPI_01)
**What’s the impact?**<br><br>The benefits and challenges of using the online forum.	**Confidence in PPI**	Confidence in their ability to do, and the benefits of, PPI.	“I felt a bit more confident that I'd made further attempt to engage members of the public, because it's quite important for my research that I do that” (RSR_02)
	**Inclusivity**	Opportunities for including people who may not be involved otherwise	“But you can now capture people from all over the country, who will not, and could not, and wouldn’t want to make the train journey from Norfolk, Preston or Scotland to get to King’s” (PPI_02)
	**Keeping up to date**	Using the forum to stay up to date with work at the Institute	“I look at it occasionally, just to see what’s going on, if you like.” (PPI_01)
	**Feedback**	Using the forum for feedback (e.g. on documents, research methods, etc.)	“I think the feedback I've had face-to-face has been much better than online, but I don't think that necessarily needs to be the case, but I think just in my limited experience of the forum, I think if I get into it more and used it more that that could change.” (RSR_01)
	**Sharing experiences**	Opportunity to share personal experiences	“I just went on the forum and I wasn't even sure if this was the purpose. I just had a little bit of a laying down how sad it was. There were two deaths that were very poorly managed for the family and both of you thanked me and my intention was, obviously to get it off my chest but to just point out to researchers there's still so much to do. We've got such a big gap to fill in educating professionals about a good death.” (PPI_04)
